# TRACP‐5b/BAP Score After 3 Months of Treatment With Combined SERM/E2 Therapy Can Predict Changes in Lumbar Spine Bone Mineral Density After 1 Year of Treatment in Early Postmenopausal Osteopenia

**DOI:** 10.1002/jbm4.10690

**Published:** 2022-10-20

**Authors:** Ikuko Ota, Yoshiaki Ota, Kuniaki Ota, Homare Eda, Hiroaki Ohta

**Affiliations:** ^1^ Department of Gynecology Kurashiki Heisei Hospital Okayama Japan; ^2^ Department of Gynecological Oncology Kawasaki Medical School Okayama Japan; ^3^ Department of Obstetrics and Gynecology Tokyo Rosai Hospital, Japan Labour Health and Safety Organization Tokyo Japan; ^4^ Department of Orthopaedic Surgery, Graduate School of Medicine Chiba University Chiba Japan; ^5^ Department of Obstetrics and Gynecology 2 Kawasaki Medical School Okayama Japan

**Keywords:** ANTIRESORPTIVES, BIOCHEMICAL MARKERS OF BONE TURNOVER, BONE REMODELING, MENOPAUSE, OSTEOPOROSIS

## Abstract

Although changes in bone mineral density (BMD) are important indexes in osteoporosis treatment, no markers are available to predict them. Given the importance of assessing the therapeutic windows of antiresorptives, we explored potential biomarkers of bone remodeling in patients receiving treatment for osteopenia. Postmenopausal women with osteopenia (defined as a lumbar BMD *T*‐score <−1.0 standard deviation (SD) below that of a reference population but >−2.5 SD) were administered estradiol 1 mg/d and bazedoxifene 20 mg/d. After 3 months of treatment, we evaluated their ratio of serum bone‐specific tartrate‐resistant acid phosphatase to bone‐specific alkaline phosphatase (TRACP‐5b/BAP), which is widely used for evaluating bone turnover in postmenopausal patients with osteoporosis in Japan because their minimum significant changes are smaller than other bone turnover markers such as carboxy‐terminal collagen cross‐links (CTX) or N‐terminal propeptide of type I procollagen (P1NP) and thus, accurately reflect bone turnover. After 1 year of treatment, we assessed changes in lumbar BMD. The cut‐off TRACP‐5b/BAP scores for a ≤−2% decrease and ≥2% increase in lumbar spine BMD were 38.4 and 29.0, respectively. The TRACP‐5b/BAP scores were associated with significantly greater areas under the curve than the other evaluated parameters. These results suggest that the TRACP‐5b/BAP score after 3 months of osteopenia treatment can predict changes in lumbar BMD after 1 year of treatment. Moreover, a receiver operating characteristic curve analysis of TRACP‐5b/BAP scores after 3 months of antiresorptive therapy and percent changes in BMD at 1 year revealed that the TRACP‐5b/BAP score, as an index of the balance between bone resorption and formation markers, has the potential to serve as a modulator of the anabolic window reflective of bone remodeling. This study's findings also suggested a role for TRACP‐5b/BAP score as a predictor of a non‐response to antiresorptive therapy, thus offering health economic implications for osteoporosis treatment. © 2022 The Authors. *JBMR Plus* published by Wiley Periodicals LLC on behalf of American Society for Bone and Mineral Research.

## Introduction

During menopause, declining estrogen levels lead to accelerated bone loss, thus increasing a woman's risk of osteoporosis and osteoporotic fracture,^(^ ^1^, ^2^ ^)^ with the latter in particular associated with increased mortality and significant morbidity, such as pain, loss of mobility, height loss, spinal deformity, and an increased need for long‐term care.^(^ ^2^ ^)^ Hormone therapy (HT), the most effective treatment for postmenopausal women with menopausal symptoms,^(^ ^3^ ^)^ effectively prevents postmenopausal bone loss^(^ ^2^, ^3^, ^4^ ^)^ and fractures, even in non‐osteoporotic women.^(^ ^1^, ^2^, ^5^ ^)^

Although HT has conventionally involved combination therapy of estrogen and progestogen (EPT),^(^ ^1^, ^2^ ^)^ there are safety concerns over EPT consisting of progestin and medroxyprogesterone acetate, such as an increased risk of breast cancer, venous thromboembolism (VTE), and stroke.^(^ ^1^, ^6^, ^7^, ^8^ ^)^ Because successful osteoporosis prevention may require long‐term therapy, these EPT‐associated risks are of particular concern,^(^ ^9^ ^)^ and the search for alternatives for postmenopausal women that provide the benefits of HT with fewer risks led to the development of a tissue‐selective estrogen complex (TSEC), a combination of one or more estrogens with a selective estrogen‐receptor modulator (SERM) designed to optimize the tissue‐selective activity of each component.^(^ ^10^, ^11^ ^)^ An ideal TSEC is expected to prevent postmenopausal bone loss as well as other menopausal symptoms without stimulating the breast and uterine tissues.^(^ ^10^, ^11^, ^12^ ^)^ TSEC has also been shown to decrease bone turnover and bone loss in postmenopausal women at increased risk of developing osteoporosis.^(^ ^13^ ^)^

Of note, because of the increasing aging of the Japanese society, while most prevalent in women in their 90s,^(^ ^14^ ^)^ hip fractures are also increasing in women in their 50s, suggesting that they remain inadequately treated and that treating women with osteoporosis will likely reduce the incidence of osteoporotic fractures, thus reducing the associated medical costs.^(^ ^15^ ^)^ In women in their 50s with a long life expectancy, osteoporotic fractures, particularly hip fractures that adversely affect their healthy life expectancy, remain an extremely important issue for which counteracting reductions in BMD is deemed vitally important to ensuring their lifelong bone health.

In its most recent position statement,^(^ ^16^ ^)^ the North American Menopause Society (NAMS) recommended pharmacotherapy for postmenopausal women, with TSEC treatment listed among the effective interventions.^(^ ^17^ ^)^ TSEC treatments are widely used worldwide; however, to our knowledge, no study to date has predicted their effects on changes in BMD.

Of the bone turnover markers (BTMs) available for monitoring the effects of osteoporosis treatment, the International Osteoporosis Foundation recommends the use of procollagen type I N‐propeptide (s‐PINP) for bone formation and serum carboxy‐terminal cross‐linking telopeptide of type I collagen (s‐CTX) for bone resorption as reference standards.^(^ ^18^ ^)^ In this study of postmenopausal women receiving osteoporosis treatment, serum bone‐specific tartrate‐resistant acid phosphatase (TRAP‐5b) and bone‐specific alkaline phosphatase (BAP), which are widely used in Japan because their minimum significant changes are smaller than other BTMs such as CTX or P1NP,^(^ ^19^ ^)^ were used as BTMs for bone resorption and formation markers, respectively. Again, the TRACP‐5b/BAP score reflects changes in BMD more sharply than either alone as a convenient and simplified uncoupling index that is readily available for clinical use.

Therefore, this study aimed to explore approaches to detect changes in BMD as early as possible in postmenopausal patients receiving TSEC. The study endpoint was the change in the bone formation marker BAP and the bone resorption marker TRACP‐5b at 3 months after TSEC initiation. Here we report the results of our study, in which a combination of these markers was shown to predict future changes in BMD.

## Materials and Methods

### Participants

This study included a total of 421 women within 3 years of menopause onset (mean age, 55.6 ± 3.4 years) being treated at Outpatient Gynecology Clinic, Kurashiki Heisei Hospital, Okayama, Japan, in 2008 to 2016 whose TRACP‐5b values were 420 mU/dL or higher due to osteopenia (−2.5 SD < lumbar BMD *T*‐score < −1.0 SD). Participants were judged to have menopause if they were ≥40 years old and had had no menstrual cycles for 1 year or if their measured follicle‐stimulating hormone (FSH) values were 40 mIU/mL or higher and their 17β‐estradiol (E2) values were 20 pg/mL or lower. This study excluded patients in whom the use of the study drug was deemed contraindicated; those who presented with a low BMD due to secondary osteoporosis or other causes; those with serious complications such as cardiovascular, hepatic, or renal disease; and those with a current or prior history of osteoporosis treatment. The institutional review board of Kurashiki Heisei Hospital approved the study, and all eligible patients provided written informed consent to participate in the study before its initiation and received started treatment with bazedoxifene (BZA; 20 mg/d) and E2 (1 mg/d) for osteopenia. The study incorporated a 1‐month washout period after treatment with selective estrogen receptor modulators and a 6‐month washout period after treatment with bisphosphonates based on the previous randomized cohort trial of bisphosphonate and SERM.^(^ ^20^ ^)^ This study was registered in the UMIN Clinical Trial Registry, and written informed consent was obtained from all patients before randomization.

### Measurements

All eligible patients underwent lumbar (L_1_ to L_4_) BMD measurements using dual‐energy X‐ray absorptiometry (Discovery A, Hologic Inc., Bedford, MA, USA) at baseline and after 1 year of BZA/E2 treatment. In addition to E2 and FSH levels, BAP (μg/L) and TRACP‐5b (mU/dL) values were measured with a chemiluminescent enzyme immunoassay and an enzyme‐linked immunosorbent assay, respectively, at the initial visit and after 3 months of E2/BZA treatment at SRL Inc (Tokyo, Japan) according to the available BZA clinical trial data regarding changes in BTMs^(^ ^21^ ^)^ (Table 1). Participants were assigned to groups according to their BMD change (≤2% decrease, <2% decrease or <2% increase, and ≥2% increase in lumbar spine BMD) for analysis. These cut‐off values were determined in accordance with the dual‐energy X‐ray absorptiometry protocol and based on previously reported reductions in BMD after the onset of menopause.^(^ ^22^ ^)^

**Table 1:** Patient Characteristics

	Treatment group lumbar BMD (−2.5 SD < *T*‐score <−1.0 SD)
	*n* = 421
Age (years)	55.6 **±** 3.4
Body mass index (kg/m^2^)	23.9 **±** 5.1
Time since menopause (years)	2.2 ± 1.4
Lumbar (L_1_ to L_4_) BMD (g/cm^2^)	0.756 ± 0.21
E_2_ (pg/mL)	<20.0
FSH (mIU/mL)	124.5 **±** 25.6
TRACP‐5b (mU/dL)	643 **±** 87
BAP (μg/L)	18.4 **±** 3.4
TRACP‐5b value after 3 months of treatment (mU/dL)	273 **±** 56
BAP value after 3 months of treatment (μg/L)	12.1 **±** 2.7

BMD = bone mineral density; FSH = follicle‐stimulating hormone; TRACP‐5b = bone‐specific tartrate‐resistant acid phosphatase; BAP = bone‐specific alkaline phosphatase.

Data are presented as mean ± standard deviation (SD).

The relevant hormone and lipid values were measured of all participants, who underwent annual screening examinations for breast and uterine cancer.

### Statistical analysis

Statistical analyses were performed using SPSS software for Windows version 26.0 (IBM Corp, Armonk, NY, USA). The receiver operating characteristic (ROC) curve analysis was employed as appropriate. Differences were considered statistically significant at *p* < 0.05.

## Results

During the first year, 6.9% of the women (29/421) discontinued the study because of failure to present to the clinic. Of the 392 patients, 170 (43.4%) showed an increase in BMD of 2% or more, 132 (33.7%) showed a decrease of 2% or more, and 90 (23.0%) showed an increase or decrease of less than 2%. During the treatment period, endometrial hyperplasia, severe hot flushes, atrophic vaginitis, or breast pain was not observed. Furthermore, no adverse events, such as obstructive vascular disease of the arteries and veins, were observed.

**Fig. 1:**
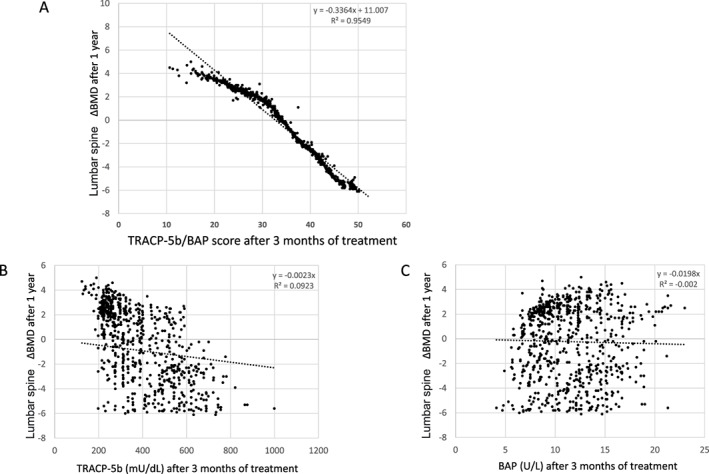
Correlation between tartrate‐resistant acid phosphatase to bone‐specific alkaline phosphatase (TRACP‐5b/BAP) score after 3 months of treatment and percent changes in lumbar bone mineral density (BMD; *T*‐score) after 1 year of treatment. Linear approximations were calculated, and TRACP‐5b/BAP scores (*A*), TRACP‐5b (*B*), and BAP (*C*) after 3 months of treatment were calculated from the percentage changes in lumbar BMD (*T*‐score) after 1 year of treatment using linear approximations.

After 3 months of treatment, their TRACP‐5b values had decreased by more than 40% from baseline, and their BAP values had decreased by more than 65%, a larger decrease than that observed in the TRACP‐5b values (Table 1). All participants were also evaluated for TRACP‐5b/BAP scores after 3 months of treatment as well as changes in lumbar BMD (percent changes in lumbar BMD) after 1 year of treatment. These correlations were assessed using the linear approximation formula: *y* = −0.3364*x* + 11.007 (*R* ^2^ = 0.95), and the TRACP‐5b/BAP scores were 26.8, 32.7, and 41.6 for BMD changes of 2%, 0%, and −2%, respectively (Fig. 1*A*). On the other hand, no significant correlation was observed between TRACP‐5b values after 3 months of treatment and changes in lumbar BMD after 1 year of treatment (Fig. 1*B*) and BAP values after 3 months of treatment and changes in lumber BMD after 1 year of treatment (Fig. 1*C*).

**Fig. 2:**
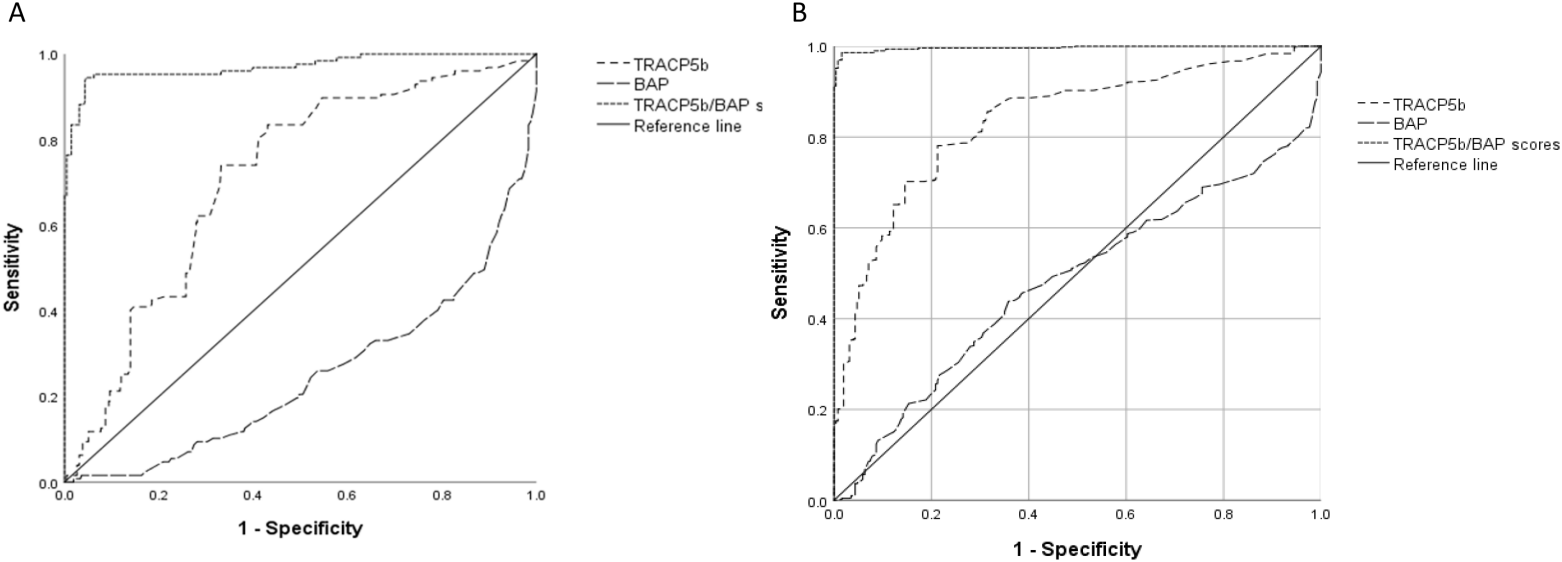
(*A*) Receiver operating characteristic curves for tartrate‐resistant acid phosphatase to bone‐specific alkaline phosphatase (TRACP‐5b/BAP) scores and percent changes in lumbar bone mineral density (BMD; *T*‐score). Cut‐off TRACP‐5b/BAP scores were associated with lumbar BMD *T*‐score ≤−2% at 1 year (*n* = 132). The cut‐off values for TRACP‐5b/BAP score and TRACP‐5b were 38.4 (sensitivity 0.986; specificity 0.984) and 308.5 mU/dL (sensitivity 0.992; specificity 0.423), respectively. (*B*) Receiver operating characteristic curves for TRACP‐5b/BAP scores and percent changes in lumbar BMD (*T*‐score). Cut‐off TRACP‐5b/BAP scores were associated with a 1‐year lumbar BMD *T*‐score ≥2% (*n* = 170). The cut‐off values for TRACP‐5b/BAP score and TRACP‐5b were 29.0 (sensitivity 0.965; specificity 0.541) and 297.5 mU/dL (sensitivity 0.787; specificity 0.736), respectively.

The ROC curves were also calculated for TRACP‐5b/BAP scores, TRAC‐5b values, and BAP values after 3 months of treatment to derive cut‐offs for 1‐year percent changes in lumbar BMD as ≤−2% (*n* = 132) (Fig. 2*A*).

Of the factors evaluated, those significantly associated with a 1‐year lumbar BMD *T*‐score ≤−2% were TRACP‐5b/BAP score (area under the ROC curve [AUC] 0.971; 95% confidence interval [CI] 0.952–0.989; *p* < 0.0001) and the TRACP‐5b value (AUC 0.711; 95% CI 0.662–0.759; *p* < 0.001), with TRACP‐5b/BAP score and TRACP‐5b cut‐offs being 38.4 (sensitivity, 0.986; specificity, 0.984) and 308.5 mU/dL (sensitivity, 0.992; specificity, 0.423), respectively (Fig. 2*A*).

The factors significantly associated with a 1‐year lumbar BMD *T*‐score ≥2% (*n* = 170) were TRACP‐5b/BAP score (AUC 0.996; *p* < 0.05; 95% CI 0.993–0.999) and TRACP‐5b value (AUC 0.836; *p* < 0.05; 95% CI 0.807–0.866), with TRACP‐5b/BAP score and TRACP‐5b cut‐offs being 29.0 (sensitivity 0.965; specificity 0.541) and 297.5 mU/dL (sensitivity 0787; specificity 0.736), respectively (Fig. 2*B*).

Furthermore, of the predictors of changes in lumbar BMD at 1 year, the TRACP‐5b/BAP score after 3 months of treatment had a significantly larger AUC and higher sensitivity and specificity than the TRACP‐5b value after 3 months of treatment. In contrast, serum BAP level after 3 months of treatment was not correlated with lumbar BMD.

## Discussion

This study included a cohort of consecutive gynecological outpatients and demonstrated that the TRACP‐5b/BAP score after 3 months of treatment reflects changes in lumbar BMD after 1 year of treatment with a significantly larger AUC than the other evaluated markers. The serum TRACP‐5b/BAP score after 3 months of treatment was a strong independent predictor of bone loss and a cut‐off‐dependent predictor of a bone mass increase. It also demonstrated greater discriminatory precision than the other laboratory parameters.

Although the IFCC/IOF Bone Marker Standards Working Party BTMs recommend the use of serum CTX and serum P1NP as BTMs,^(^ ^18^ ^)^ we used TRACP‐5b as a bone resorption marker and BAP as a bone formation marker in this study. The extracellular secretion of TRACP‐5b, an enzyme derived from osteoclasts in bone tissue that degrades within a few days that is widely used in Japan as a BTM, from osteoclasts is shown to increase in response to elevated bone resorption by osteoclast activation.^(^ ^23^ ^)^ Importantly, the minimum significant changes of TRACP‐5b and BAP are smaller than other BTMs, including CTX or P1NP.^(^ ^19^ ^)^ This is because TRACP‐5b and BAP are not peptides like CTX or P1NP, which are easily affected by several factors in blood but not the enzyme activity; furthermore, unlike CTX, they are less influenced by renal function. Thus, these markers were employed to correctly estimate the bone resorption and formation status of postmenopausal women.

However, BTMs vary even among premenopausal women depending on their age and other factors, making it difficult to establish their normal values.^(^ ^24^ ^)^ In fact, although numerous studies have evaluated the prognostic value of BTMs, they have yielded conflicting results, ^(^ ^25^, ^26^, ^27^, ^28^, ^29^, ^30^, ^31^, ^32^, ^33^ ^)^ and BTMs are now recommended only for monitoring osteoporosis treatment efficacy and compliance.^(^ ^25^, ^34^, ^35^ ^)^ However, it is difficult to determine the state of bone metabolism from a single bone resorption or formation marker value alone given that bone remodeling does not always remain balanced, especially in postmenopausal women.^(^ ^36^ ^)^ Moreover, varying therapeutic windows for antiresorptives have been reported in terms of differences in suppressing bone resorption versus formation, thus variably contributing to BMD increases.^(^ ^37^, ^38^ ^)^ These reports show that the different effects of different osteoporosis treatments on BMD are due to their differential effects on bone remodeling, suggesting the importance of accurately evaluating bone remodeling during osteoporosis treatment.

Therefore, here we focused on the therapeutic window as a balance between bone formation and resorption and hypothesized that changes in bone turnover are more closely reflected by TRACP‐5b/BAP score than any single BTM. Importantly, the TRACP‐5b/BAP score evaluated at one time point during treatment revealed the participants' bone turnover status and predicted changes in BMD after 1 year of treatment. In this study, the TRACP‐5b/BAP score was evaluated after 3 months of treatment according to the available BZA clinical trial data regarding changes in BTMs.^(^ ^21^ ^)^ These results indicate that the TRACP‐5b/BAP score may be a more reliable and simpler index than any single BTM, which requires pre‐ and post‐treatment measurements. Although no prior studies have used TRACP‐5b/BAP score, our results are consistent with those of previous studies showing that bone balance correlated weakly with BMD changes^(^ ^39^ ^)^; the procollagen type 1 N‐terminal peptide/β CTX (P1NP/β CTX) ratio was associated with osteoporotic nonvertebral fractures in older adults^(^ ^40^ ^)^; and the urinary resorption marker u‐cross‐linked N‐telopeptide of type I collagen/the serum bone formation marker osteocalcin (NTX/OC) ratio was predictive of fractures independent of the Fracture Risk Assessment Tool.^(^ ^41^ ^)^ Importantly, the TRACP‐5b/BAP score after 3 months of treatment showed a much stronger correlation with BMD changes after 1 year of treatment (*R* ^2^ = 0.95) than any index ever evaluated, the reason being that TRACP‐5b/BAP score could reflect bone metabolism more accurately than CTX, NTX, or OC because minimum significant changes of TRACP‐5b and BAP are smaller than other BTMs. Our study employed a formula whose denominator and numerator were represented by a bone formation marker and a bone resorption marker unlike that used in an earlier study by Fisher and colleagues (procollagen type 1 N‐terminal peptide/β CTX [P1NP/β CTX])^(^ ^40^ ^)^ because the bone resorption marker, designated as the numerator, was assumed to reflect changes in response to antiresorptive use.

Importantly, our results showed that the TRACP‐5b/BAP score after 3 months of treatment had a significantly larger AUC and higher sensitivity and specificity than TRACP‐5b after 3 months of treatment alone, whereas serum BAP after 3 months of treatment showed no correlation with lumbar BMD after 1 year of treatment, suggesting the importance of accurately evaluating bone remodeling using the ratio of the bone formation marker to the bone resorption marker. It is also suggested that given its higher sensitivity and specificity, the TRACP‐5b/BAP score has the potential to predict changes in lumbar BMD associated with antiresorptive therapy more sharply than TRACP‐5b alone.

This is the first study to demonstrate the significance of TRACP‐5b/BAP score, that is, the ratio of the bone resorption marker TRACP‐5b to the bone formation marker BAP; minimum significant changes of both are smaller than other BTMs. Thus, TRACP‐5b/BAP score appears to be a viable marker that accurately captures the bone remodeling status.

This study had some limitations. First, how TRACP‐5b/BAP score behaves over time after the start of antiresorptive therapy with agents other than E2 and BZA remains unclear, and further studies are needed to determine the optimal timing(s) for its measurement. Second, the study data were derived from a single center and the participating postmenopausal women were all Japanese, thus limiting the generalizability of our findings. Finally, BMD changes were used as the study endpoint and may not truly reflect changes in fracture risk.

In conclusion, given the importance of assessing the therapeutic window of antiresorptives during osteoporosis treatment as well as their inhibitory effects on bone resorption or their stimulatory effects on bone formation, this study investigated whether TRACP‐5b/BAP score might prove useful in determining the therapeutic window of each osteoporosis treatment and predicting BMD changes after treatment. A ROC analysis of TRACP‐5b/BAP scores after 3 months of antiresorptive therapy and percent changes in BMD at 1 year revealed that TRACP‐5b/BAP score is a sufficiently sensitive marker with a large AUC that can reflect lumbar BMD changes. Thus, the TRACP‐5b/BAP score, an index of the balance between bone resorption and formation markers, has the potential to modulate the anabolic window reflective of bone remodeling. Furthermore, the study's findings also suggested a role of TRACP‐5b/BAP score as a predictor of non‐response to antiresorptive therapy, thus offering health economic implications for osteoporosis treatment.

## Disclosures

IO, YO, KO, and HO declare no conflicts of interest in association with this study. HE is an employee of Pfizer Japan Inc.

## Author Contributions

**Ikuko Ota:** Investigation; methodology; visualization; writing – review and editing. **Yoshiaki Ota:** Data curation; investigation; methodology; validation; writing – review and editing. **Kuniaki Ota:** Investigation; methodology; validation; writing – review and editing. **Homare Eda:** Data curation; formal analysis; investigation; writing – original draft; writing – review and editing. **Hiroaki Ohta:** Conceptualization; investigation; project administration; supervision; writing – original draft.

### Peer Review

The peer review history for this article is available at https://publons.com/publon/10.1002/jbm4.10690.

## Data Availability

The data that support the findings of this study are available from the corresponding author upon reasonable request.
